# Estimating spatial accessibility to facilities on the regional scale: an extended commuting-based interaction potential model

**DOI:** 10.1186/1476-072X-10-2

**Published:** 2011-01-10

**Authors:** Paul Salze, Arnaud Banos, Jean-Michel Oppert, Hélène Charreire, Romain Casey, Chantal Simon, Basile Chaix, Dominique Badariotti, Christiane Weber

**Affiliations:** 1 Université de Strasbourg; Image, Ville, Environnement, Strasbourg, France; 2 CNRS, ERL 7230, Strasbourg, France; 3 UMR 8504, CNRS/Université Paris 1, Géographie-Cités, Paris, France; 4 UREN, INSERM U557/INRA U1125/CNAM/Université Paris 13/CRNH Ile-de-France, Bobigny, France; 5 Université Pierre et Marie Curie-Paris6; Service de Nutrition, Groupe Hospitalier Pitié-Salpêtrière (AP-HP), Centre de Recherche en Nutrition Humaine Ile-de-France (CRNH IdF), Paris, France; 6 Lab-Urba, Institut d'Urbanisme de Paris, Université Paris Est-Créteil, France; 7 Université de Lyon, INSERM/U870 INRA/U1235, CRNH Rhône-Alpes, Hospices Civils de Lyon, Oullins, France; 8 INSERM U707, Paris, France; 9 Université Pierre et Marie Curie-Paris6, UMR-S 707, Paris, France

## Abstract

**Background:**

There is growing interest in the study of the relationships between individual health-related behaviours (e.g. food intake and physical activity) and measurements of spatial accessibility to the associated facilities (e.g. food outlets and sport facilities). The aim of this study is to propose measurements of spatial accessibility to facilities on the regional scale, using aggregated data. We first used a potential accessibility model that partly makes it possible to overcome the limitations of the most frequently used indices such as the count of opportunities within a given neighbourhood. We then propose an extended model in order to take into account both home and work-based accessibility for a commuting population.

**Results:**

Potential accessibility estimation provides a very different picture of the accessibility levels experienced by the population than the more classical "number of opportunities per census tract" index. The extended model for commuters increases the overall accessibility levels but this increase differs according to the urbanisation level. Strongest increases are observed in some rural municipalities with initial low accessibility levels. Distance to major urban poles seems to play an essential role.

**Conclusions:**

Accessibility is a multi-dimensional concept that should integrate some aspects of travel behaviour. Our work supports the evidence that the choice of appropriate accessibility indices including both residential and non-residential environmental features is necessary. Such models have potential implications for providing relevant information to policy-makers in the field of public health.

## Background

### Measuring spatial accessibility

Accessibility is a major issue for many types of stakeholders in policy making in the fields of transport, urban planning, marketing and public health. Because it may encompass more dimensions than the spatial one (e.g. temporal, social, economic), there is no single established definition of accessibility. Several literature reviews provide a global and historical overview of existing definitions and associated measures, as well as some developments and examples of applications [1-7]. A useful classification of the existing operational accessibility measures has been proposed by Geurs and van Wee [7]. The authors distinguish four broad categories of measurements. "Infrastructure-based" measurements are used to assess the efficiency of the transport network (e.g. traffic congestion, mean travel speed). "Location-based" measurements deal with the spatial distribution of opportunities (e.g. distance to the nearest opportunity, number of available facilities within a neighbourhood), generally at an aggregated level. "Person-based" measurements refer to disaggregated space-time accessibility measurements at the individual level. "Utility-based" measurements are based on benefits assessment and utility maximisation theory for both individuals and population groups. Whatever the category, specifying the measurement makes it necessary to define some interrelated elements: the degree and type of disaggregation, origins and destinations, attractiveness and travel impedance [6].

In the public health domain, there is growing interest in the study of the relationships between individual health behaviours (e.g. food intake, physical activity) and measurements of spatial accessibility to the related opportunities (e.g. food outlets, sport facilities) [8,9]. One important aim is to assess whether social deprivation is associated with specific spatial accessibility levels to certain types of facilities, contributing to an amplification of the social disparities in unhealthy behaviours [10].

In a recent methodological review, we noted that in most previous studies, spatial accessibility to a given type of facilities was measured either as the distance to the nearest opportunity or as a count or density of opportunities within a neighbourhood (administrative unit or time/distance buffer) [11]. Although these "classical" measurements are very useful due to their simplicity (both to understand and to compute), they present some limitations. Indeed, by ignoring some aspects of travel behaviour, they only provide a "one-dimensional" biased view of accessibility [12].

### The limits of "classical" indices

The nearest opportunity measurement assumes that surrounding opportunities other than the nearest one are not included in the possible destinations that individuals may choose. Handy and Niemeier [6] have shown that this is an unrealistic assumption. Indeed, in two communities in the San Francisco Bay Area (CA, USA), they found that more than 80% of the residents used to visit more than one supermarket in a month.

The count of facilities within a neighbourhood, also known as a *container *index [12], overcomes this limitation by considering all available opportunities within a neighbourhood. However, it assumes that an opportunity situated just beyond the limit of the neighbourhood will not be accessible and that all the opportunities within a neighbourhood are equally accessible, which is questionable with respect to spatial barriers or the perception of the distances.

In order to address this last question, the use of kernel density estimation (KDE) [13] and of an enhanced two-step floating catchment area method (E2SFCA) [14] have been proposed to assess accessibility to health care [14-16] or food stores/physical activity facilities [17-19]. The main idea of such methods is to take into account both the demand (population) and the supply (health practitioners) side and to partly include travel impedance specification (frictional effect of space: more weight is given to opportunities near to the origin). Nevertheless, most of these studies used the distance weighting function provided by the available GIS software without addressing that specific point.

The delimitation of the neighbourhood [20] in *container *index, KDE and E2SFCA method is another critical point. Using circular or network-based buffers instead of administrative units may be more appropriate because it frees the study from administrative boundaries. Unfortunately this approach does not solve the issue of "clear-cut neighbourhood boundaries" and the choice of the size and shape of the buffer remains problematic [21]. This last point about neighbourhood delimitation and the fact that accessibility to facilities can be seen as environmental exposure [22] naturally lead us to the broader question of how the environment is to be defined.

### Defining the environment

By focusing on residential neighbourhoods and ignoring potential exposure that occurs around other activity places (e.g. workplace or school), most studies in the health literature have fallen into what has been called the "residential trap" [23]. In some studies, exposure or accessibility levels have been assessed around schools [24-27] or both homes and workplaces [28]. In this study, origins (i.e. workplace and home) were considered separately when assessing relationships between accessibility and health outcomes and it would have been interesting to focus on cumulative exposure. While the "residential trap" is no longer relevant (because not only homes are taken into account) it could be more appropriate to see this problem as the absence of a dynamic dimension ("motionless trap").

### Including the dynamic dimension of accessibility

It appears to be necessary to consider both residential and non-residential environmental influences on health behaviours, which implies including spatial or spatial-temporal dynamics of individuals and populations (i.e. mobility). In that sense, "person-based" or disaggregated individual-space-time measurements of accessibility [29] are totally relevant but the results may be difficult to interpret for population-wide studies. For example, they make it possible to evaluate accessibility levels over a whole day in regard to location and duration of activities according to individual characteristics (e.g. gender) [30]. In health studies, Kestens and colleagues [31] used individual experienced activity spaces to measure accessibility to different kinds of food stores in each location visited during a weekday by different categories of population (according to age and income). Such methods require large sets of very detailed data which are not always available, especially for large study zones. That point is discussed by the authors who used data from a very large mobility survey. However, because of limited information on time use, they were unable to integrate temporal constraints (i.e. time-budgets) in their estimations of accessibility levels.

### Aim of the study

The general aim of the work was to propose measurements of accessibility to a set of facilities (three types of food outlet: hyper/supermarkets, grocery stores, bakeries) on the regional scale. In a context of generalised car-owning and thus increasing accessibility levels, more and more people chose to live in suburban and rural areas (growing suburbanisation process) which are associated with better living environments. The functioning of urban, suburban and rural areas cannot be disconnected from each other and have then to be seen as a whole system, making the regional scale a level of particular interest.

Because of the aggregated nature of available data and in order to overcome some of the limitations of the measurements mentioned above (nearest opportunity, *container *index, KDE), we chose to use a potential accessibility index [32]. Accessibility is defined as a potential for spatial interaction (i.e. an intensity of possible destinations) that makes it possible to take account of a global aspect of travel behaviour.

In the first part of this work, we present some historical and theoretical considerations and then provide a complete example of application of a potential model including a detailed calibration process. The second part of the paper is dedicated to the presentation and application of an extended potential model for a commuting population.

## Methods

### Study zone

**Figure 1:**
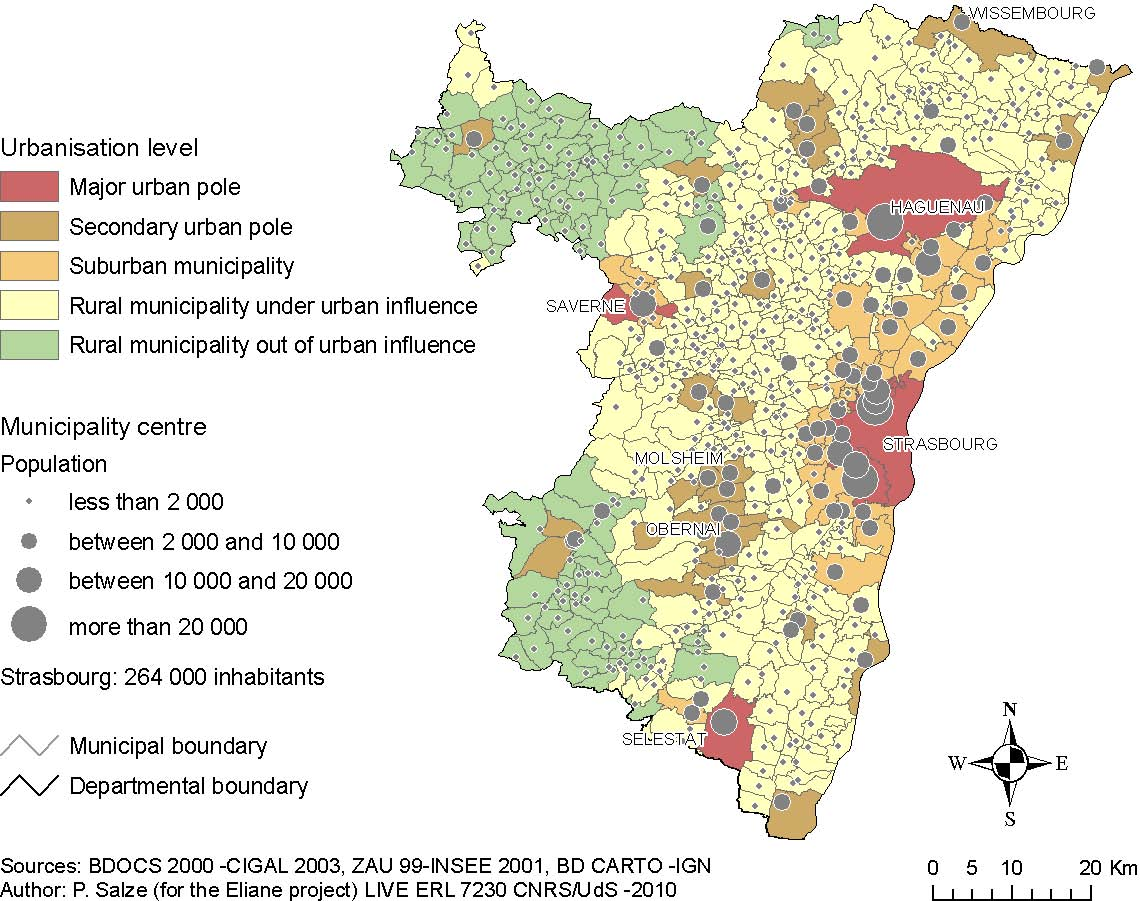
**Bas-Rhin *département*: urbanisation level and population**. This map shows the distribution of population and urbanisation levels in the Bas-Rhin *département*. About half of the population of the *département *lives in Greater Strasbourg (i.e. Strasbourg city and 27 surrounding municipalities). Other important cities are Haguenau (32,000 inhabitants) and Sélestat (17,000 inhabitants). Most of the *département *exhibits low urbanisation levels: 446 out of 526 municipalities have less than 2000 inhabitants.

Our study territory was the Bas-Rhin *département*, an administrative region of about 4800 km^2 ^situated in Eastern France. Greater Strasbourg (Strasbourg city and surrounding municipalities) is the main regional metropolis, accounting for about 50% of the population of the *département*. Built upon land-use, demographic and employment data, Figure 1 provides a general overview of the extent of urban, suburban and rural areas in the *département *[33]. Regarding urbanisation levels, it is important to note that our study area is fairly heterogeneous, resulting in marked spatial disparities in the distribution of population, facilities, services and work opportunities, and thus in expected accessibility levels.

### Data on distribution of facilities

Data on the distribution of facilities were obtained from the French National Institute of Statistics and Economic Studies (INSEE). Two censuses, the Municipal Census (Inventaire Communal, 1998) and the Businesses Census (SIRENE, 2000) provide the number of available opportunities for each administrative unit (526 municipalities in the Bas-Rhin *département*). In this work, we have chosen to focus on three types of food outlet (bakeries, grocery stores and hyper/supermarkets) that may present contrasting situations. Bakeries and grocery stores can be seen as proximity services and both grocery stores and hyper/supermarkets sell general food items, but have retail floor areas of less and more than 120 m^2 ^respectively (1998 Municipal Census classification). Table 1 shows that variability in the distribution of food outlets across the territory was high and that the shopping behaviours of French households differed according to the type of food outlet [34].

**Table 1:** Distribution of food outlets among municipalities of the Bas-Rhin *département *(France) according to number and type of food outlets

Number of food outlets	Type of food outlet		
	
	Bakeries	Grocery stores	Hyper/supermarkets
0	250 (47.5)	344 (65.4)	456 (86.7)
1 - 2	219 (41.6)	166 (31.6)	49 (9.3)
3 - 5	41 (7.8)	11 (2.1)	18 (3.4)
6 - 7	5 (1.0)	3 (0.6)	2 (0.4)
8 - 10	7 (1.3)	1 (0.2)	0 (0.0)
More than 10	4 (0.8)	1 (0.2)	1 (0.2)
% of households shopping at least once a week	65	14	83
Mean number of visits in a week	3.7	1.8	2.0

In the upper part of the table, values are number of municipalities with percentages in parentheses. In the lower part, values given for grocery stores include little supermarkets (retail floor area between 120 and 300 m^2^).

**Figure 2:**
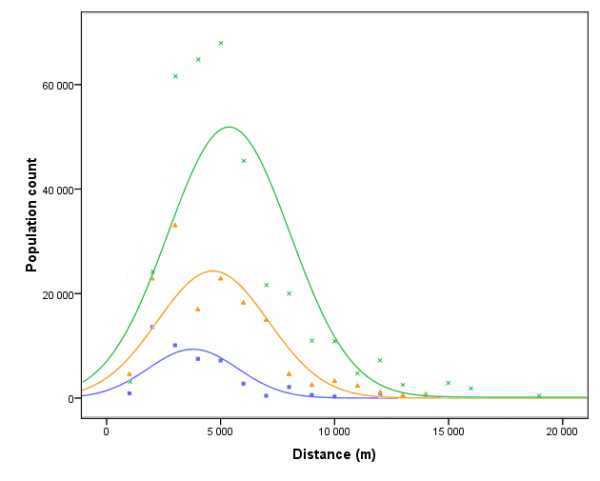
**Distribution of trip length for shopping purposes**. The graph shows population counts by trip length (1 km intervals) for observed travels to hyper/supermarkets (green), grocery stores (orange) and bakeries (blue). Trip lengths are Euclidean distances between administrative centres of municipalities. Dots represent observed values; lines are Gaussian probability density functions. Trip lengths distribution shows a bell-shaped pattern with an under-representation of population counts for short distances. This can be due to the absence of data for intra-urban travels and a possible spatial structure effect (i.e. relatively few centres of municipalities situated less than 2 km away one from the other).

The 1998 Municipal Census provides additional data on travel behaviour: for each type of opportunity, if it is not available in a given municipality, the destination (i.e. the municipality) chosen by the majority of inhabitants is provided. Even if it is incomplete because of the absence of data for intra-municipality and extra-*département *trips, that information makes it possible to approach the distribution of spatial interactions (Figure 2).

### The potential model as an accessibility index

It is generally recognized that the use of potential models as accessibility indices was first introduced by Hansen [32]. The potential model belongs to the family of gravity-based or spatial interaction models. These models are based on social physics and assume some analogies between physical (e.g. Newton's Law of Gravitation) and social phenomena such as migration [35], retailing [36] or population distribution [37].

The concept of potential first appeared in Stewart [37] who noted that the influence of population between two places was inversely proportional to the distance between them (inverse distance weighting function). Since those early years, many other forms of distance weighting functions have been proposed for spatial interaction models in general, and for potential models more specifically (e.g. inverse power, negative exponential). As stated by Pooler [[38], p. 276], "virtually any function which is monotonically decreasing with increasing *d*_*ij *_is a candidate for inclusion in a potential equation". A general formulation of the potential model can then be written as:

(1) $$\Phi_{i}^{s}=\sum_{j=1}^{n}O_{j}^{s}\cdot f(d_{ij})$$

where $\Phi_{i}^{s}$ is the potential at point *i *for a given type of opportunity *s*, $O_{j}^{s}$ is an opportunity at *j*, *d*_*ij *_is the distance (or time) between *i *and *j *and *f(d*_*ij*_*) *is an impedance travel (or distance decay) function for travel between *i *and *j*. It can refer to travel behaviour or "frictional effect of space" and can be seen as people's willingness to travel according to trip purpose (e.g. to go shopping for food or to go to a fitness centre for performing physical activity), demographic characteristics (e.g. age, gender) or destination attractiveness.

Inverse power $f(d_{ij})=d_{ij}^{-\gamma }$ and negative exponential *f*(*d*_*ij*_) = exp(-*α*·*d*_*ij*_) are the most commonly used functions. According to Handy and Niemeier [[6], p.1177], the latter is "the most closely tied to travel behaviour theory". These functions have been implemented in the Accessibility Analyst extension for the desktop GIS software package Arc View 3 [39]. However, we will show below that the use of other functions may be relevant.

It is important to note that some analogy exists between KDE and potential model. In both cases, opportunities are weighted according to a function of the distance (gravity-based models). The main difference between the two methods lies in the mathematical properties of the function. For KDE, the kernel is a function that integrates to one while it is not necessarily the case for the potential model. This allows a greater degree of flexibility in the definition of the type of function and of the associated parameters.

### Model calibration

In our work, the calibration process consisted in defining two elements of the model specification: the travel impedance and the set of potential destinations. In order to specify travel impedance, three steps were necessary: 1) choosing the distance metric *d*_*ij *_(e.g. Euclidean distance, travel time), 2) defining the travel impedance function *f(d*_*ij*_*) *(e.g. inverse power, negative exponential) and 3) setting the parameters of this function (e.g. the constant). These stages are presented in the next three sections. The specification of the set of potential destinations (i.e. neighbourhood delimitation) is presented in a fourth section.

### Choosing the distance metric

One critical point when estimating spatial accessibility concerns the definition of the distance measurement [40]. Many different "distances" may be used including Euclidean distance, Manhattan (or rectangular) distance, network distance, time-distance or economic cost. Because using sophisticated and more precise measures such as travel times may introduce computational difficulties, we sought to verify whether simpler Euclidean distances would be very different from travel times or network distances on the regional scale.

We first built and validated a model in order to estimate travel times between all the municipalities in the *département *(see Appendix 1 for details). Pearson correlation coefficients were then calculated in order to assess the strength of the associations between Euclidean distance, network distance and time-distance of observed commuting trips (N = 4690). Data were log-transformed because of data distribution skewness. Results showed that the associations between all three measures were very strong (correlations above 0.97), so that we could conclude that Euclidean distances were a good approximation of the two other more specific distances for the regional scale. We therefore decided to keep our models as simple as possible by using only Euclidean distances.

### Defining a travel impedance function

In order to define the travel impedance function, Taylor's proposal [41] was to find a linear relation between trip length and volume of interactions. For this purpose, he suggested transforming data according to the Goux typology of distance decay functions (i.e. square-root exponential, exponential, normal, Pareto and log-normal) and to find which of these transformations provides the best fit (i.e. least squares).

**Figure 3:**
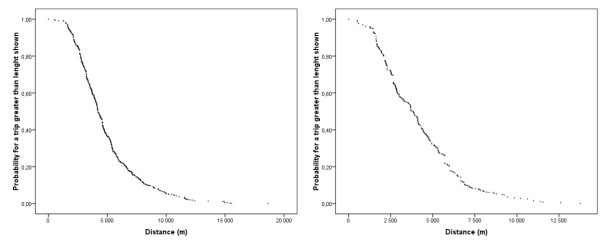
**Distribution of probabilities for spatial interaction at a given distance**. These graphs show cumulated per cent of trips that are greater than a given distance for hyper/supermarkets (left) and grocery stores (right). Data for bakeries are not shown. Plotted values can also be seen as the probability for interaction at a given distance. It allowed us to have an idea of the type of distance decay function.

We applied this method to available data on trip lengths for shopping purposes (Figure 2). Because none of the transformations allowed us to get an acceptable linear pattern, we adopted a probabilistic approach [[42,43] cited by Ingram [1]]. This led us to represent data differently (Figure 3) and we found that the distance weighting function would belong to a generalised negative exponential functions family. This family of functions is defined as:

(2) $$f(d_{ij})=\exp(-\alpha \cdot d_{ij}{}^{\beta })$$

where *β *is a distance exponent to be determined. The Gaussian function is a particular case for which the distance exponent equals two. Smoothing properties (convexo-concave shape) of this family of functions make it "related to empirical results referring both to the perception of space and to the mobility of populations" [[44], p.14].

### Setting the parameters for the travel impedance function

Once the type of function was defined, the next step consisted in setting the associated parameters (i.e. *α *and *β*) that would produce the best fit to the observed data [41]. That can be done by performing either linear or non-linear regression analysis with transformed or non-transformed data respectively. In the case of linear regression, the idea is to conduct the analysis for different values of the distance exponent *β*.

Both methods were tested (see Appendix 2) and produced equivalent goodness of fit (R^2 ^= 0.99; p < 0.001). Nevertheless, by plotting observed data and the predicted values of the regression model, some differences were found (results not shown): the non-linear regression model tightly fit observed data for intermediate distances but seemed to underestimate probabilities for shorter and longer trips. In contrast, the linear regression model seemed to better estimate probabilities for longer trips but clearly underestimated values for short and intermediate distances.

That was particularly true in the case of hyper/supermarkets and our conclusion for this point was that probability values predicted by the linear regression model were closer to the idea we have about the phenomenon (e. g. for hyper/supermarkets, a probability value for spatial interaction that tends towards zero below 10 km does not seem to be realistic, see Figure 2). Consequently, we decided to calibrate the travel impedance function for each type of food outlet using the values of the parameters derived from the linear regression analyses (Table 2).

**Table 2:** Parameters values retained from the calibration process

	Parameter value		Span (in m)
		
	α	β	
Bakeries	2.14.10^-6^	1.6	12 000
grocery stores	9.333.10^-7^	1.65	14 000
Hyper/supermarkets	1.156.10^-6^	1.6	19 000

### Specifying the set of potential destinations

Because no spatial interactions are observed beyond a certain distance threshold for a given purpose (e.g. 20 km for hyper/supermarkets, see Figure 2) and because of the asymptotic nature of the exponential function (i.e. every opportunity of the study would contribute to the potential value calculation), it may be necessary to define a maximum distance *Dij *(or span) above which opportunities would not be included in the calculation (i.e. defining neighbourhood limits).

Because the negative exponential function is short-tailed, long distances have limited effects on the accessibility estimation, and function truncation thus does not lead to an important loss of information [44]. New formulation of the potential can then be written as:

(3) $$\Phi_{i}^{s}=\{\begin{array}{ll} \sum_{j=1}^{n}O_{j}^{s}\cdot f(d_{ij}) & \text{if }d_{ij}\leq D_{ij}\\ 0 & \text{otherwise} \end{array}$$

Obviously, this new formulation is to be related to the *container *approach (rectangular distance decay function) [5] but it overcomes its limitation (a rough threshold) if the impedance travel function is correctly calibrated and tends towards zero when *d*_*ij *_is close to the span value *Dij*. In the present work, spans were defined according to the distribution of trip lengths (Figure 2) as the maximum travel distance observed rounded to the higher integer value in kilometres (see Table 2).

## Results

### Potential accessibility estimation: application of the "original" potential model

Using the calibrated model, we estimated potential values for each type of food outlet in the study zone. The models were implemented in the XLISP-STAT programming environment [45,46] and ArcGIS 9.2 (ESRI, Redlands, California) was used for mapping.

One of the advantages of the potential model is that it can be used as a spatial smoothing technique [42] and allows producing pseudo-continuous (raster data) surface maps when applied to a regular grid of points. We applied this transformation from discrete to pseudo-continuous by estimating potential accessibility values for the whole *département *(with a 5 km margin for border effect correction) on a regular grid of more than 1,000,000 of points (spatial resolution: 100 m). This was the finest spatial resolution we could process with reasonable computing times. Once the accessibility values had been estimated for each type of food outlet, outputs of the models were mapped by converting point values into raster data with the 100 m spatial resolution.

**Figure 4:**
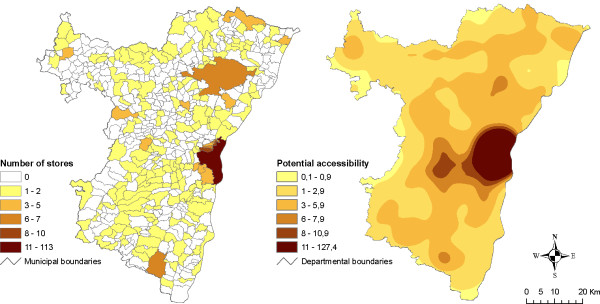
**Maps of number of grocery stores (left) and potential accessibility surface (right)**. These maps show the distribution of grocery stores by municipality (left) and smoothed surface of potential accessibility (right). Potential accessibility was estimated with an exponential-shaped function and a 14 km span. Class limits were defined manually for visualisation and comparison purposes.

**Figure 5:**
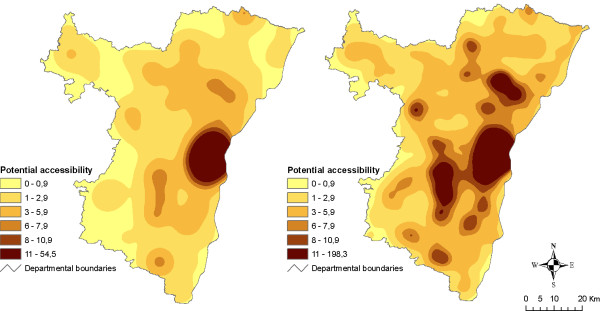
**Potential accessibility surfaces for hyper/supermarkets (left) and bakeries (right)**. These maps show smoothed surfaces of potential accessibility to hyper/supermarkets (left) and bakeries (right). Potential accessibility was estimated with an exponential-shaped function and a 19 km span for hyper/supermarkets and a 12 km span for bakeries. Class limits were defined manually for visualisation and comparison purposes.

Results are presented on Figures 4 and 5. Producing pseudo-continuous surface maps presents at least two main advantages. First, by getting data that are free from administrative boundaries, it allows approaching a more realistic and precise estimation of accessibility levels. For example, map comparison clearly shows that there are no areas with null accessibility even though facilities are not locally available (i.e. no grocery stores in the municipality) (Figure 4). Second, it proves its usefulness for emphasising different kinds of retailing strategies. For example, it appears that while accessibility to bakeries is quite good all over the *département*, hyper/supermarkets are concentrated in most populated areas (Figure 5).

### Comparing count of facilities data and potential accessibility values

**Figure 6:**
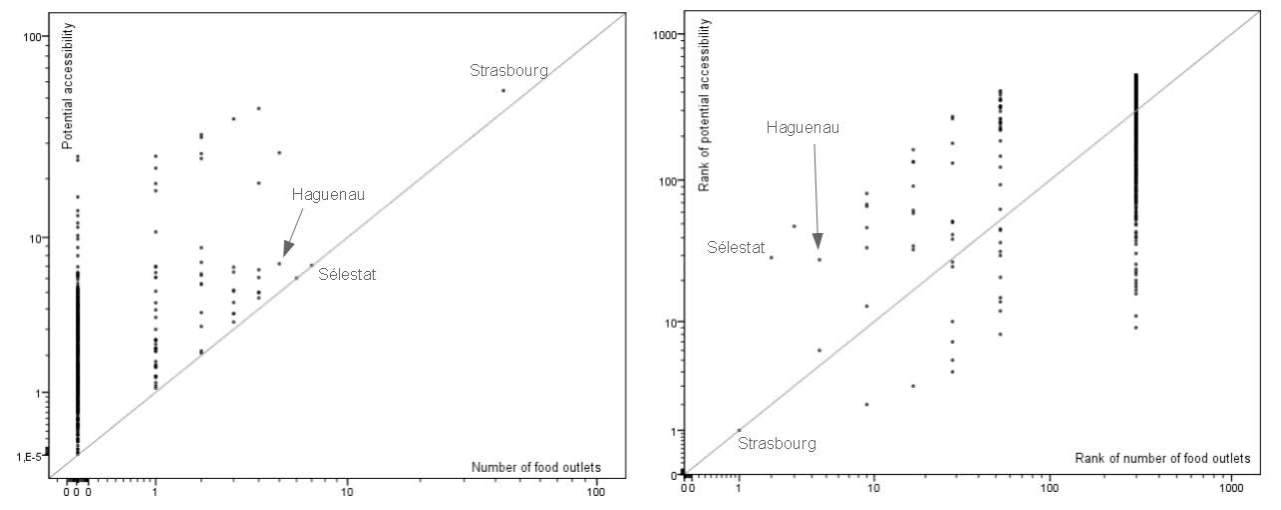
**Graphical comparisons between original data and model's output**. The graphs show the differences between frequencies of hyper/supermarkets and potential accessibility values (on the left) and ranks (on the right). Line equation is y = x.

In order to compare the output of the first model with original data, potential accessibility values were estimated for all municipalities administrative centres (N = 526). Graphical comparisons were conducted between potential accessibility values and number of food outlets and between ranking of municipalities (first rank for the highest values) according to potential accessibility values and count of facilities data. In both cases, results showed a very high variability in the outputs of the model: except for Strasbourg city, which continued to occupy the first place, ranking of municipalities appeared completely modified, even for important cities such as Haguenau and Sélestat (Figure 6). Correlation coefficients were estimated in order to assess the importance of these variations. Because of the extreme skewness of the original data distribution (see Table 1), we estimated Spearman's rank correlation coefficients. All associations were statistically significant at the 0.01 level. Results indicate a poor relationship between the rankings of municipalities for the count of facilities and potential accessibility values (0.342 for grocery stores, 0.355 for hyper/supermarkets and 0.523 for bakeries).

### An extended potential model for commuters

The approach developed previously allowed us to estimate accessibility levels, but in a very limited and static way. Indeed, so far we did not take into account population movements across space while the presence of opportunities both in the residential neighbourhood and around activity places (e.g. workplaces) may impact population accessibility levels.

One basic idea is therefore to extend the potential model in order to take account of the case of commuters: people have access to opportunities not only in the area *i *where they reside, but also in the area *k *where they work.

In that case, we can estimate a cumulative potential accessibility to a type of service *s *such as:

(4) $$\Phi_{Cik}^{s}=\Phi_{Cik,i}^{s}+\Phi_{Cik,k}^{s}$$

where $\Phi_{Cik,i}^{s}$ and $\Phi_{Cik,k}^{s}$ are respectively the potential accessibility in *i and k *for commuters living in *i *and working in *k*.

This first approach does not consider trip length *d*_*ik *_between *i *(home) and *k *(work) and hence that increasing travel time will reduce available time (or time-budget) for a given purpose (e.g. shopping). Because time-budget is not unlimited, if commuting time becomes too long, commuters will not have access to services neither in *i *nor *k*. It is then necessary to choose a threshold value for trip length beyond which facilities will not be accessible anymore. This threshold *max(d*_*ik*_*) *can be defined in an empirical way (e.g. using commuting data), assuming for example that the time-budget for activities is null when commuting time or distance reaches the threshold value. The available time-budget for a given activity in *i *and *k *will be reduced according to a trip length weighting function:

(5) $$\gamma (d_{ik})={\left(\frac{d_{ik}}{\max(d_{ik})}\right)}^{\theta },\;d_{ik}\leq \max(d_{ik})$$

where *θ *is a parameter reflecting the relationship between the commuting time or distance and the available time-budget. Standardisation by *max(d*_*ik*_*) *ensures that *γ(d*_*ik*_*) *ranges from zero when *d*_*ik *_equals zero (i.e. living and working in the same place) to one when *d*_*ik *_equals *max(d*_*ik*_*)*.

The potential accessibility $\Phi_{Cik}^{s}$ to a service *s *for commuters living in *i *and working in *k *can then be written as:

(6) $$\Phi_{Cik}^{s}=(\Phi_{Cik,i}^{s}+\Phi_{Cik,k}^{s})\cdot (1-\gamma (d_{ik}))$$

where (1 - *γ*(*d*_*ik*_)) is the trip length weighting term which ranges from zero (when no time is available for the given activity, i.e. the travel time is too long) to one (when full time-budget is available, i.e. no travel time). $\Phi_{Cik}^{s}$ then theoretically ranges from 0 to $2\times \Phi_{Cik,i}^{s}$ when *i *and *k *coincide (i.e. living and working in the same place).

Because commuters of a given municipality may have different destinations, we introduce the proportion of commuters for each destination in the previous equation (Equation 6). A global cumulative potential accessibility for commuters living in a municipality *i *can then be written as:

(7) $$\Phi_{Ci}^{s}=\sum_{k=1}^{n}\frac{C_{ik}}{C_{i}}(\Phi_{Cik,i}^{s}+\Phi_{Cik,k}^{s})\cdot (1-\gamma (d_{ik}))$$

where *C*_*ik *_is the number of commuters living in *i *and working in each destination *k *and *C*_*i *_is the total number of commuters. $\Phi_{Cik,i}^{s}$ and $\Phi_{Cik,k}^{s}$ are estimated using the potential model (Equation 3).

### Estimating potential accessibility for a commuting population

The analysis conducted for the choice of the distance metric (see section "Methods: choosing the distance metric") also allowed us to calibrate the *θ *parameter in the trip length weighting function *γ*(*d*_*ik*_) (Equation 5): the high correlation coefficient value between time and distance led us to conclude that the time-budget should decrease in a linear way as the Euclidean distance increases and *θ *was then set to 1. The threshold distance for which potential accessibility value around home and work reaches zero was defined as the maximum travel distance observed for all commuters (65 km). We estimated commuting-based potential accessibility to hyper/supermarkets, grocery stores and bakeries and compared it to the outputs of the potential model. Models were calibrated using the same parameters values as previously (see Table 2).

**Figure 7:**
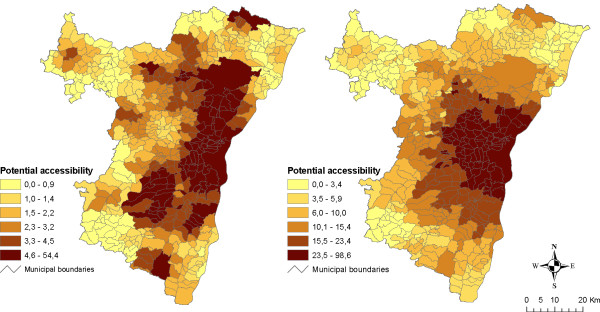
**Maps of potential accessibility to hyper/supermarkets for residents (left) and commuters (right)**. These maps make it possible to compare potential accessibility to hyper/supermarkets between residents (left) and commuters (right). Potential accessibility was estimated with an exponential-shaped function and a 19 km span. In the case of commuters, potential accessibility is cumulated in municipalities of both residence and workplace and weighted according to the inverse distance between them and the number of commuters. Class limits are defined according to quantiles.

**Figure 8:**
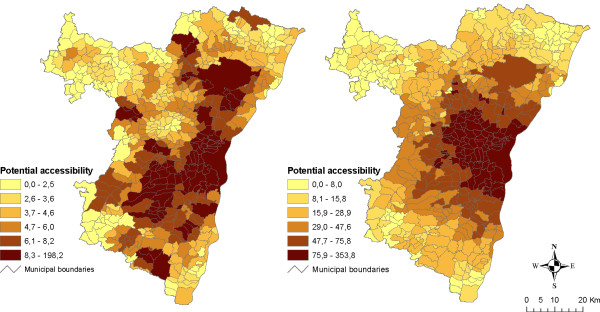
**Maps of potential accessibility to bakeries for residents (left) and commuters (right)**. These maps make it possible to compare potential accessibility to hyper/supermarkets between residents (left) and commuters (right). Potential accessibility was estimated with an exponential-shaped function and a 12 km span. In the case of commuters, potential accessibility is cumulated in municipalities of both residence and workplace and weighted according to the inverse distance between them and the number of commuters. Class limits are defined according to quantiles.

Cartographic outputs for hyper/supermarkets and bakeries are presented in Figures 7 and 8. Because our application is based upon municipality-level data (commuting data) accessibility values are then estimated for administrative centres but mapped for municipality areas. Map analysis shows that the introduction of journey to work into the model dramatically increases the overall accessibility levels. The impact of Strasbourg city as the biggest employment centre is particularly striking. Indeed in both cases, values seem to follow a clear inverse gradient as the distance to Strasbourg city increases.

### Comparing potential accessibility between residents and commuters

**Figure 9:**
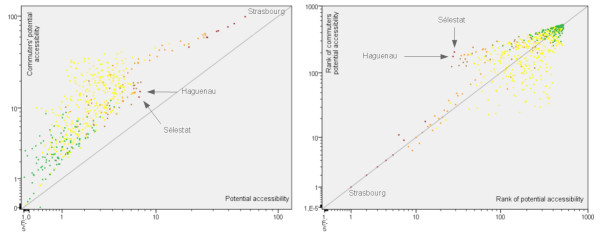
**Potential accessibility to hyper/supermarkets for residents and commuters: graphical comparisons**. The graphs show the differences between values (on the left) and ranks (on the right) of potential accessibility to hyper/supermarkets according to urbanisation levels. Major urban poles are plotted in red, secondary poles in brown, suburban municipalities in orange and rural municipalities under or out of urban influence respectively in yellow and green. Line equation is y = x.

**Figure 10:**
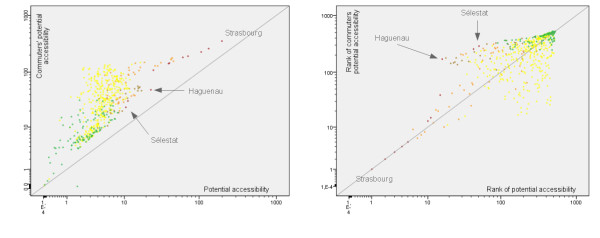
**Potential accessibility to bakeries for residents and commuters: graphical comparisons**. The graphs show the differences between values (on the left) and ranks (on the right) of potential accessibility to bakeries according to urbanisation levels. Major urban poles are plotted in red, secondary poles in brown, suburban municipalities in orange and rural municipalities under or out of urban influence respectively in yellow and green. Line equation is y = x.

Graphical comparisons reinforce the previous observations of an overall increase in accessibility levels either for supermarkets (Figure 9, left), bakeries (Figure 10, left) or grocery stores (results not shown). In all cases, the increase is high in major urban poles and suburban municipalities. The situation is more contrasted in the case of rural municipalities under urban influence: the increase is very strong for some of them while others have similar levels as rural municipalities out of urban influence. Graphical comparison of ranks confirms that observation (Figure 9 and 10, right) and Spearman's rank correlation coefficients show that the ranking variability is slightly higher for bakeries (0.665) and grocery stores (0.674) than for hyper/supermarkets (0.778). These results can be related to the previous map analysis: distance to urban poles plays a major role in the estimation of accessibility levels. That seems to be especially true in rural remote areas where accessibility levels for both residents and commuters are the lowest.

## Discussion

The objective of this study was to propose measures of spatial accessibility to a set of facilities on the regional scale, with the aim of applying the methodology to the field of health behaviours and neighbourhood deprivation in future work. For this purpose, we first chose to use a potential accessibility index, and we proposed to improve it by introducing the dynamics of a commuting population.

### Data availability

Our developments have mainly been driven by available data. Because commuting data were only available at an aggregated level, it has not been possible to conduct the analysis below the municipality level. Furthermore, as we decided to work with a fairly old database, we were unable to assess data quality (see for example [47], [48] and [49] for methodological discussions on this point). In the present work, we chose to apply our models to three different kinds of food outlets for illustrative purposes, and it is important to emphasise here that the method could be applied to any type of facility (e.g. public services, sport facilities) and using disaggregate data if available.

### The potential model: introducing travel behaviour

Our work was based on the observation that most studies that attempted to link health behaviours and spatial accessibility used measurements that do not take into account travel behaviour (nearest opportunity, container index) [11]. We exposed a complete example of application (including calibration process) of a potential model which is relatively easy to implement and for which only few data are needed for computation. The potential model is very close to the KDE both in a theoretical (gravity-based measure) and practical (spatial smoothing technique) perspective. The main advantage of our method is that every part of the model specification can be controlled, especially the definition of the travel impedance function. This advantage is nevertheless very relative as it is widely accepted that in the case of KDE, compared to the type of function used, the size of the span is much more crucial (i.e. neighbourhood delimitation) [13].

The resulting potential accessibility index includes some aspects of travel behaviour and is partly free from administrative boundaries. It may thus provide a more accurate picture of accessibility levels experienced by the population. In our study zone, it appeared for example that areas with null or very weak accessibility were almost inexistent, reflecting a good global accessibility level to food outlets. These results obviously need to be put in relation with the transport mode chosen and thus with car ownership and transportation possibilities. But because 85% of the households of the *département *owned at least one car (1999 Population Census, INSEE), we hypothesise that our resulting index is valid on this scale of analysis and for this specific study zone.

A classical limitation of such aggregated models is that it assumes that all individuals in a municipality experience the same accessibility level, thus the same "frictional effect of space". Indeed, the model was calibrated using trip length data, assuming that spatial interaction distributions were only due to travel behaviours, the distance decay being constant for the whole zone studied and the population homogeneous. The model thus did not take into account possible local spatial variations of the distance decay which can result either from behavioural discrepancies or from the spatial structure effect [50].

Another limitation is that of the lack of data that may have resulted in inaccurate measurements. Results showed that areas with the lowest accessibility levels were mainly distributed near the borders of our study zone (Figure 4). This observation has nevertheless to be interpreted carefully because of the absence of information about facilities provision outside the *département*. Indeed it has been shown that edge effects may strongly impact gravity-based accessibility measurements (especially when using large distances) [51].

### Integrating the spatial dynamics of a commuting population

Our proposition was to extend the potential model to a commuting population. The resulting index reflects an accessibility level by car that takes into account the cumulated spatial distribution of facilities around both home and workplace. This model resulted in an overall increase in the accessibility levels. The increase was nevertheless not uniform, and the results highlighted the role of distance to major urban poles. Some rural municipalities with low levels of accessibility ultimately enjoyed better accessibility levels than some secondary urban poles. These observations emphasise the fact that considering populations as static "objects" could lead to biased results and may partly explain why published studies on the relationships between individual behaviours and environmental determinants exhibit inconsistent findings [52]. This important result also supports the calls for the developments of new measurements taking into account both residential and non-residential exposure [22,53,23,31].

The other strength of this extended model is that it introduces a temporal constraint, although in a very coarse way, through the notion of time-budget. In their study on foodscape exposure, Kestens and colleagues identified this point as an important one in measuring the influence of food stores in a space-time perspective [31].

Several limitations are associated with the development of our extended potential model. First, the distance and time-budget weighting functions were based on Euclidean distances between municipalities because our analysis showed a very strong correlation between travel-times, network distances and Euclidean distances on the regional scale. Nevertheless, the travel-time model used (see Appendix 1) did not take account of road traffic data, which may strongly impact trip durations (especially during peak hours). Further investigation will therefore be necessary to refine the travel-time model and more generally, to address this question of the time-budget weighting function. It may be indeed relevant to disaggregate our index and to calibrate the weighting functions for different segments of population, e.g. according to median income, age and structures of households [31,54], motorisation rates [55] or travel modes [56].

A second limitation is that the model only considers accessibility for origins (municipality of residence) and destinations (workplace) and thus leads to the underestimation of potential accessibility levels because facilities present along the daily space-time path are not taken into account. Obviously, the extended model we propose can be refined to include these intervening opportunities. However, we believe with other authors [57,31] that a methodological shift towards individual-based and activity-based models would be necessary to address this question in health studies. In the meantime, given actual data availability, the use of aggregated models still remains necessary when dealing with large study zones.

## Conclusions

The aim of this study was to propose measurements of spatial accessibility to a set of facilities on a regional scale. The measurements provided different pictures of accessibility levels. We first applied a potential model which overcomes some of the limitations of more simple accessibility indices by partly encompassing the multidimensional aspect of the accessibility estimation issue [12]. Then, we proposed an extension to the original potential model. That allowed us to get very different results by integrating the dynamics of commuting. Our work supports existing evidence of the importance of the inclusion of specific questions about the location of non-residential activities in health surveys and of the choice of appropriate accessibility indices for analyses dealing with 1) socio-spatial disparities in facilities related to health behaviours and 2) relationships between individual behaviours and accessibility to specific facilities. Such extension of spatial interaction models has potential implications for improving our understanding of environmental influences on health outcomes on the regional scale, and then for providing relevant information to policy-makers in the field of public health.

## Appendix 1 - Building and validating the travel time model

Using a road network database (Georoute 2002, Institut Géographique National, France) and the Network Analyst extension for ArcGIS 9.2 (Environmental Systems Research Institute, Redlands, CA), we estimated network distances and travel times between all the municipalities in the *département*. Speed limits were assigned to each segment of the road network following previous work of Hilal [58], according to land-use type (urban vs. rural areas), elevation (hill areas vs. plain areas) and road hierarchy (highways, primary roads, secondary roads).

**Figure 11:**
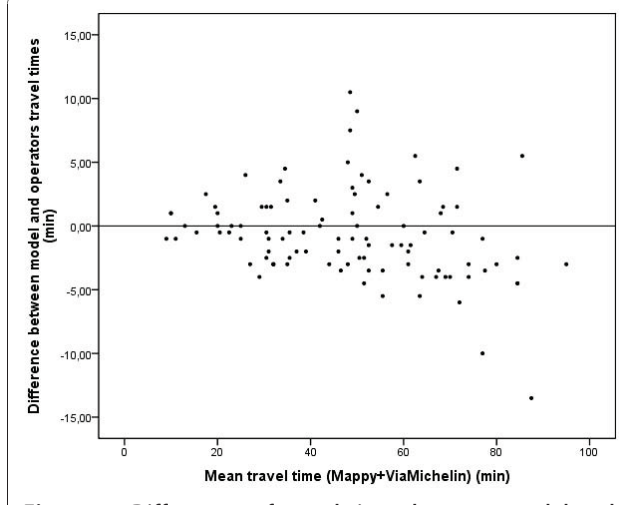
**Differences of travel times between model and operators according to trip length**. This graph shows differences between travel times estimated by our model and on-line operators. Difference in minutes is plotted for each pair of municipalities exchanging commuters according to trip duration.

This "travel-time" model was validated for 100 randomly selected journeys, by comparing calculated travel times with travel times obtained from two different on-line mapping/itinerary providers (^© ^Mappy and ^© ^ViaMichelin). Student's paired t-tests were used to assess the differences between travel times. Results showed that the differences between travel times were statistically significant for each of the three tested pairs (p < 0.001). Interestingly, we observed that, compared to the travel times provided by our model, data from the first operator tended to overestimate and those from the second operator tended to underestimate travel times. A Student's paired t-test was then applied to assess the differences between the travel times of the model and the mean of the two travel times provided by the operators. Even though very close to the threshold value, the test did not reach statistical significance (N = 100; p = 0.052). Furthermore, 95% of the travel times difference absolute values were lower than 7.4 minutes (mean: 2.71; SD: 2.32) and the largest travel times differences (above 5 minutes) were observed for the larger trip lengths (above 50 minutes) (Figure 11).

## Appendix 2 - Setting the parameters of the distance weighting function

**Figure 12:**
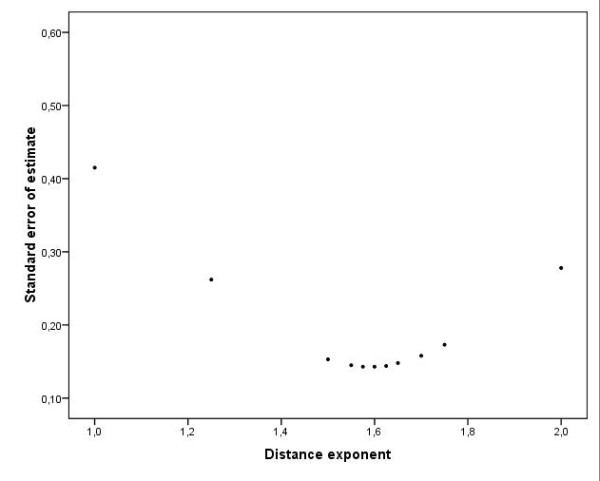
**Standard error of estimates of linear regression model for hyper/supermarkets according to distance exponent**. This graph shows standard error of estimates resulting from the linear regression model according to several distance exponents.

In a first step, data were log-transformed and linear regression analyses performed for several distance exponents in order to find the best fit (i.e. the minimal standard error of estimate) (Figure 12). Because of our probabilistic approach (probability reaches 1 at null distance), the constant term was not included in the regression model which then took the form $\log\;(P_{i})=-\alpha \cdot d_{ij}{}^{\beta }$ where *P*_*i *_is the probability for interaction at distance *d*_*ij*_.

For non-linear regression analyses, the model was of the form $P_{i}=\exp\;(-\alpha \cdot d_{ij}{}^{\beta })$ and the initial parameters used in the iterative process have been set to values relatively close to expected ones (i.e. rounded values of parameters derived from the linear regression analysis).

## Competing interests

The authors declare that they have no competing interests.

## Authors' contributions

PS and AB conceived and designed the study. PS performed analysis and interpretation of data, parts of the programming and drafted the manuscript. AB performed the main part of the programming and helped to draft the manuscript. JMO, HC, CS, BC, RC, DB, and CW participated in the critical revision of manuscript. All authors have read and approved the final manuscript.
